# Microbial metabolite sodium butyrate enhances the anti-tumor efficacy of 5-fluorouracil against colorectal cancer by modulating PINK1/Parkin signaling and intestinal flora

**DOI:** 10.1038/s41598-024-63993-x

**Published:** 2024-06-06

**Authors:** Yangbo Li, Pengzhan He, Ying Chen, Jiaming Hu, Beiying Deng, Chuan Liu, Baoping Yu, Weiguo Dong

**Affiliations:** 1 Department of Gastroenterology, Renmin Hospital of Wuhan University, Wuhan, 430060 Hubei Province People’s Republic of China; 2 Central Laboratory, Renmin Hospital of Wuhan University, Wuhan, People’s Republic of China

**Keywords:** Sodium butyrate, Colorectal cancer, Apoptosis, Reactive oxygen species, PINK1/Parkin, Cancer, Microbiology, Gastroenterology, Oncology

## Abstract

Colorectal cancer (CRC) is a prevalent global health issue, with 5-fluorouracil (5-FU) being a commonly used chemotherapeutic agent for its treatment. However, the efficacy of 5-FU is often hindered by drug tolerance. Sodium butyrate (NaB), a derivative of intestinal flora, has demonstrated anti-cancer properties both in vitro and in vivo through pro-apoptotic effects and has shown promise in improving outcomes when used in conjunction with traditional chemotherapy agents. This study seeks to evaluate the impact and potential mechanisms of NaB in combination with 5-FU on CRC. We employed a comprehensive set of assays, including CCK-8, EdU staining, Hoechst 33258 staining, flow cytometry, ROS assay, MMP assay, immunofluorescence, and mitophagy assay, to detect the effect of NaB on the biological function of CRC cells in vitro. Western blotting and immunohistochemistry were used to verify the above experimental results. The xenograft tumor model was established to evaluate the in vivo anti-CRC activity of NaB. Subsequently, 16S rRNA gene sequencing was used to analyze the intestinal flora. The findings of our study demonstrate that sodium butyrate (NaB) exerts inhibitory effects on tumor cell proliferation and promotes tumor cell apoptosis in vitro, while also impeding tumor progression in vivo through the enhancement of the mitophagy pathway. Furthermore, the combined treatment of NaB and 5-fluorouracil (5-FU) yielded superior therapeutic outcomes compared to monotherapy with either agent. Moreover, this combination therapy resulted in the specific enrichment of Bacteroides, LigiLactobacillus, butyric acid-producing bacteria, and acetic acid-producing bacteria in the intestinal microbiota. The improvement in the intestinal microbiota contributed to enhanced therapeutic outcomes and reduced the adverse effects of 5-FU. Taken together, these findings indicate that NaB, a histone acetylation inhibitor synthesized through intestinal flora fermentation, has the potential to significantly enhance the therapeutic efficacy of 5-FU in CRC treatment and improve the prognosis of CRC patients.

## Introduction

Colorectal cancer (CRC) remains a prominent cause of cancer-related fatalities and holds the third position in the global landscape of malignancy incidence^1^. Presently, the primary therapeutic modalities for CRC entail a synergistic amalgamation of surgical intervention and chemotherapeutic intervention, wherein 5-fluorouracil (5-FU) predominates as the principal pharmaceutical agent of election in the majority of instances. Notably, the emergence of drug resistance, particularly pertaining to 5-FU and docetaxel, widely acknowledged as conventional therapeutic modalities, has precipitated a progressive deterioration in the efficacy of curative measures^2^. Furthermore, it is noteworthy that the administration of 5-FU may perturb the composition of the gastrointestinal microbiota, thereby engendering perturbations in the gut barrier integrity and fostering an inflammatory milieu within the colon^3–5^. Consequently, there arises an imperative to identify novel, naturally-derived anticancer drugs characterized by minimal toxicity and heightened efficacy to design new combinations of chemotherapy regimens to prevent the worsening of drug resistance.

The intestinal microbiome assumes a pivotal role in upholding gut homeostasis, safeguarding integrity of the mucus barrier, orchestrating the host immune responses, mediating medication toxicity, and influencing the response to anticancer pharmaceutical agents^6^. Sodium butyrate (NaB), a derivative of short-chain fatty acids (SCFAs), emerges as a metabolite engendered through the enzymatic degradation of dietary fiber remnants by the intestinal microflora^7^. Notably, NaB not only exerts regulatory control over intestinal functionality, bestowing vitality upon intestinal epithelial cells, and governing the composition of the microbial milieu, but also serves as an inflammation inhibitor, thereby preserving the equilibrium of the intestinal environment^8–10^. In addition, NaB is one of the natural histone deacetylase inhibitors (HDACis), a novel class of chemotherapy agents in oncology, evincing augmented efficacy and mitigated toxicity when administered in conjunction with classical therapeutic modalities^11,12^. Scholars have shown experimentally that NaB possesses the capacity to impede the proliferation and foster the apoptotic demise of an array of neoplastic cell types, including but not limited to endometrial cancer^13^, lung cancer^14^, and bladder cancer^15^. Encouragingly, NaB sensitizes neoplastic cells to the antineoplastic properties of docetaxel^16^. Crucially, NaB curtails tumor progression through a myriad of mechanisms, with particular emphasis on pathways affiliated with mitochondria^17,18^. Mitochondria, being integral to cellular viability, orchestrate a multitude of physiological processes, spanning from energy generation and metabolic regulation to signal transduction.^19^. Previous studies showed that NaB has the potential to augment reactive oxygen species (ROS) levels through depleting glutathione (GSH) reserves in malignant cells, as well as inducing the proapoptotic genes, BAX, BAK and BIK in gastric cancer cells, thereby engendering a proclivity toward apoptotic cell demise^20^.

Mitophagy serves as a pivotal mechanism for the targeted degradation of impaired mitochondria, employing the autophagy-lysosome pathway^21^. ROS exacerbates mitochondrial dysfunction, thereby constituting one of the pivotal triggers for the initiation of mitophagy^22^. As a selective form of autophagy, mitophagy distinctively targets damaged mitochondria which are ready for degradation. Of particular intrigue is the mitochondrial depolarization-induced PINK1/Parkin mitochondrial autophagy cascade, which orchestrates the ubiquitination of various mitochondrial outer membrane proteins^23^. Subsequently, activated Parkin fosters the interaction between microtubule-associated protein 1A/1B-light chain 3 (LC3) and p62, establishing a connection between the autophagosome and the ailing mitochondria, consequently instigating the process of mitophagy^24^. Moreover, the tumor suppressor proteins PINK1 and Parkin were reduced in expression in CRC compared to normal colorectal tissue^25,26^. Besides ubiquitination, the core mitophagy machinery would experience wide acetylation modification^27^. For example, deletion or inhibition of the mitochondrial deacetylase Sirtuin family proteins leads to an increase in the production of ROS. Additionally, it causes the recruitment of Parkin to mitochondria, resulting in mitophagy^28–30^. However, the precise anti-tumorigenic effects of NaB in conjunction with 5-FU in the context of CRC, along with the underlying mechanistic intricacies, remain shrouded in ambiguity.

Herein, our study aimed to investigate the inhibitory effect and mechanism of antineoplastic synergy between 5-FU and NaB on colorectal cancer cells. The results indicated that NaB affected the proliferation and apoptosis of CRC cells through inducing mitochondrial dysfunction through oxidative stress. Additionally, it was discovered that NaB can enhance the effectiveness of 5-FU in suppressing CRC. This is likely due to the induction of mitophagy in a PINK1 and Parkin-dependent manner, as well as the enhancement of the overall gut microbiome composition. Therefore, the data suggest that NaB has the potential to be an anti-tumor agent and could be a valuable adjunct to chemotherapy in the treatment of CRC.

## Materials and methods

### Materials

Sodium butyrate (purity > 99%) and 5-fluorouracil (purity > 99%) were acquired from Sigma-Aldrich (St. Louis, MO, United States). Carbonyl cyanide 4-(trifluoromethoxy)phenylhydrazone(FCCP) (10 mM) and mitochondrial division inhibitor 1 (Mdivi-1) (10 mM) were obtained from MedChemExpress(Shanghai, China). *N*-acetylcysteine (NAC) (10 mM) was obtained from Beyotime(Jiangsu, China). The following antibodies were used in this study: BCL-2 (Cat No. 26593-1-AP), BAX (Cat No. 60267-1-Ig), LC3B (Cat No. 14600-1-AP), PINK1 (Cat No. 23274-1-AP), Parkin (Cat No. 66674-1-Ig), PCNA (Cat No. 10205-2-AP), p62 (Cat No. 18420-1-AP), VDAC1 (Cat No. 55259-1-AP), TOMM20 (Cat No. 66777-1-Ig), Ki67 (Cat No. 27309-1-AP), and GAPDH (Cat No. 60004-1-Ig) were obtained from Proteintech (Wuhan, China).

### Cell culture

The human colorectal cancer cell lines (HCT-116, SW-480, and DLD-1) and the normal cell line (NCM-460) were provided by the China Center for Type Culture Collection (CCTCC). The culture conditions involved maintaining the cells at 37 °C in a humidified incubator with 5% CO_2_. These cell lines were cultured in RPMI-1640 medium supplemented with 10% fetal bovine serum and a 1% solution of penicillin and streptomycin. All experiments were conducted on cells in the logarithmic growth phase and were repeated at least three times.

### Cell counting kit-8 (CCK-8)

Cell viability was assessed through the CCK-8 assay. Cells, including HCT-116, SW-480, DLD-1, and NCM-460, were seeded in 96-well plates with a density of 5 × 10^3^ cells per well and cultured for 24 h. Upon reaching 70%–80% confluence, treatments were administered, involving various concentrations of NaB (0, 1, 2, 4, 8, 16, 32, and 64 mM), 5-FU (0, 1, 2, 4, 8, 16, 32, and 64 µg/mL), or a combination of 5-FU and NaB for an additional 24 or 48 h. Following this, 10 μL of CCK-8 reagent (Biosharp) was introduced to each well, and the incubation continued at 37 °C for 2 h. The ultimate absorbance at 450 nm was measured using a microplate reader (Perkin Elmer, Waltham, MA, USA). CompuSyn software (CompuSyn Inc., Paramus, NJ, United States) was engaged to calculate combination index (CI) values for the two drugs, and CI values below 1 signify synergistic effects.

### 5-Ethynyl-20-deoxyuridine (EdU) assay

CRC cells were subjected to an assessment of DNA synthesis using the BeyoClick™ EdU-594 Kit(Beyotime). The cells were plated in a 12-well plate at a density of 2 × 10^5^ cells per well. HCT-116 cells received treatment with 5-FU (14 µg/mL), NaB (6 mM), or a combination of both, while SW-480 cells were exposed to 5-FU (8 µg/mL), NaB (7 mM), or their combination for an additional 24 h. After a 2-h incubation with EdU, cells were washed with phosphate-buffered saline (PBS) and fixed in 4% paraformaldehyde (Biosharp) for 15 min. Following permeabilization with 0.1% Triton X-100 for 15 min, the cells were incubated with a fluorescent marker solution for 30 min. Nuclear staining was performed using 4',6-diamidino-2-phenylindole solution (Beyotime). Subsequently, the cells were sealed and examined under a fluorescence microscope (BX53, Olympus).

### Hoechst 33258 staning

Cell apoptosis was assessed using the Hoechst 33,258 Staining reagent (Beyotime). HCT-116 and SW-480 cells in the logarithmic growth phase were seeded into a 12-well plate at a density of 2 × 10^5^ cells per well and incubated overnight. Subsequently, CRC cells were treated with NaB and 5-FU as detailed in the "5-Ethynyl-20-deoxyuridine (EdU) assay" section and then stained with Hoechst 33258. Fluorescence microscopy was employed to observe morphological features of apoptosis.

### Annexin V-PE/7-AAD double staining

A flow cytometer, along with Annexin V-PE/7AAD kit (BD, Franklin Lakes, NJ, United States), was employed to quantify the percentage of apoptotic cells. HCT-116 and SW-480 cells were seeded into a 6-well plate and incubated for 24 h with various treatments as detailed in the "5-Ethynyl-20-deoxyuridine (EdU) assay" section, with or without pretreatment with NAC, FCCP, or Mdivi-1 for 2 h. Following co-staining with Annexin V-PE and 7AAD in binding buffer, the cells were subjected to flow cytometry analysis. The cells were categorized into four populations according to their distinct fluorescence characteristics: necrotic cells, live cells, late apoptotic cells, and early apoptotic cells.

### Measuring the ROS levels

2ʹ,7ʹ-dichloro-fluorescein diacetate (DCFH-DA), utilized as a ROS assay reagent (Solarbio), and flow cytometry, were employed to quantify ROS levels. HCT-116 and SW-480 cells were exposed to NaB or 5-FU at different concentrations, as outlined in the "5-Ethynyl-20-deoxyuridine (EdU) assay" section, for 24 h, with or without pretreatment with NAC for 2 h. The subsequent day, fluorescence microscopy was utilized to observe the cells following a 30-min incubation with the DCFH-DA dye in the incubator.

### Measuring the mitochondrial membrane potential

Alterations in mitochondrial membrane potential (MMP) were evaluated utilizing the MMP assay reagent JC-1 (Solarbio). Following treatment of the cells, as outlined in the "Measuring the ROS Levels" section, for 24 h. On the subsequent day, fluorescence microscopy was utilized to observe the cells following a 90-min incubation with the JC-1 dye in the incubator.

### Mitophagy assay

Mitophagy in HCT-116 and SW-480 cells was detected using the Mitophagy Detection Kit(Dojindo, Kumamoto, Japan) containing Mitophagy Dye and Lyso Dye. Concisely, CRC cells were cultured in a 6-well plate and exposed to Mitophagy Dye (100 nM) for 1 h. Then the cells underwent the various treatments detailed in the "Annexin V-PE/7-AAD double staining" section for a 12-h duration. Following rinsing with the culture medium, Lyso Dye (1 μM) was introduced and allowed to incubate for an additional hour. Subsequently, PBS was employed for washing, and fluorescence images were captured using a laser confocal fluorescence microscope (FV1200, Olympus).

### Immunofluorescence(IF)

Cells were seeded into six-well plates and given drug treatment for 24 h after reaching 70–80% confluence. Cell climbing slices were then fixed in 4% paraformaldehyde for 10 min and wereincubated in 5% bovineserum albumin (BSA) for 1  h, followed by an overnight primary antibody incubation at 4 °C. The next day, slices were washed three times in PBS and incubated in a secondary antibody solution for 1 h at 37 °C. Subsequently, slices were washed four times and mounted with anti-fade agents containing 4′, 6-diamidino-2-phenylindole (DAPI). Imageswere captured using a fluorescence microscope.

### Western blotting analysis

Total protein extraction from CRC cells and nude mouse subcutaneous tumor tissue and quantification were conducted utilizing a BCA Protein Assay Kit (Beyotime). Protein separation was accomplished through electrophoresis, followed by electrotransferring. After blocking for 1 h, the membranes underwent overnight incubation with the respective primary antibodies at 4 °C, succeeded by exposure to secondary antibodies at 25 °C for 1 h. The resulting immunoreactive protein bands were visualized using an ECL imager (QuickChemi 5200, Monad, Shanghai, China). GAPDH was utilized as a control, and ImageJ software was employed for the quantification of the blots.

### Xenograft tumor models in vivo

HCT-116 cells were harvested and washed with serum-free RPMI-1640, then suspended in PBS and subsequently implanted subcutaneously into the dorsal region of male BALB/c nude mice (18–20 g, 30 days old), obtained from Shulaibao Biotechnology (Wuhan, China). Upon the tumors attaining a volume of approximately 80–160 mm^3^, the nude mice were randomly assigned to one of four groups: control; 5-FU; NaB; and combination groups (n = 6 per group). In the 5-FU and combination groups, mice were subjected to intraperitoneal injections of 5-FU (20 mg/kg) every 2 days. In contrast, the control and NaB groups received intraperitoneal injections of an equivalent volume of normal saline solution. The NaB and combination groups underwent gavage administration of NaB (150 mg/kg) every 2 days, whereas the control and 5-FU groups were gavaged with an equivalent volume of normal saline. At each treatment interval, the weight of the mice and the volume of the tumors were assessed. Tumor volume (TV) was calculated using the formula: TV (mm^3^) = d^2^ × D/2, where d represents the shortest diameter and D represents the longest diameter. Following a 24-day observation period, the mice were euthanized, and their tumors were excised and weighed. Blood samples were collected from the mice to assess hepatic and renal function, including the activation levels of ALT, AST, BUN, and Cr.

### Immunohistochemistry (IHC)

Prepare paraffin sections and subject them to a 45-min baking process. Treat with a xylene and ethanol gradient, followed by PBS washing. Block the sections by exposing them to 3% H_2_O_2_ at room temperature for 10 min and rinse with PBS. Utilize microwave-mediated antigen retrieval with a citrate buffer, followed by PBS washing. Use serum to prevent non-specific binding and eliminate excess serum by blotting with filter paper. Incubate the tissue with the specified primary antibody at 4 °C overnight, followed by PBS washing. Apply the secondary antibody to the tissue and conduct a PBS wash. Perform DAB staining and terminate the reaction with distilled water. Counterstain with hematoxylin, then proceed to dehydrate and mount the sections. Capture images of five random fields using a microscope.

### 16S rRNA gene sequencing and microbiome analysis

Following euthanasia at the termination of the treatment regimen, fecal samples from mice were gathered and stored at −80 °C, with a portion earmarked for microbiome composition analysis. The hypervariable region V3–V4 of the bacterial 16S rRNA gene was amplified with primer pairs 338F: 5'-ACTCCTACGGGAGGCAGCA-3' and 806R: 5'-GGACTACHVGGGTWTCTAAT-3'. PCR products were checked on agarose gel and purified through the Omega DNA purification kit (Omega Inc., Norcross, GA, USA). The purified PCR products were collected and the paired ends (2 × 250 bp) were performed on the Illumina Novaseq 6000 platform. The online platform BMKCloud (https://www.biocloud.net) was used to analyze the sequencing data.

### Ethics statement

The study followed the ARRIVE guidelines. Animal experiments and care strictly followed the regulations of the National Institutes of Health for the ethical treatment and use of animals in research(NIH Publication No. 80-23; revised 1978) and received approval from the Ethics Committee of Renmin Hospital of Wuhan University (Protocol No. WDRM20221105A).

### Statistical analysis

WB and immunofluorescence images were quantized using FIJI ImageJ software based on pixel intensity (https://imagej.net/software/fiji). Co-localization analysis and calculation of Pearson's R values were conducted using the coloc-2 plugin. Statistical analyses were conducted using GraphPad Prism 8.4.3 (GraphPad Software Inc., La Jolla, CA, USA). The data were expressed as mean ± SD, and Student's t-test and one-way ANOVA were used for significance testing. Tukey's multiple comparison post hoc testwas used for comparisons between groups when appropriate. A significance threshold of *p* < 0.05 was applied to ascertain statistical significance.

## Results

### NaB combined with 5-FU inhibited the proliferation of CRC cells

**Figure 1:**
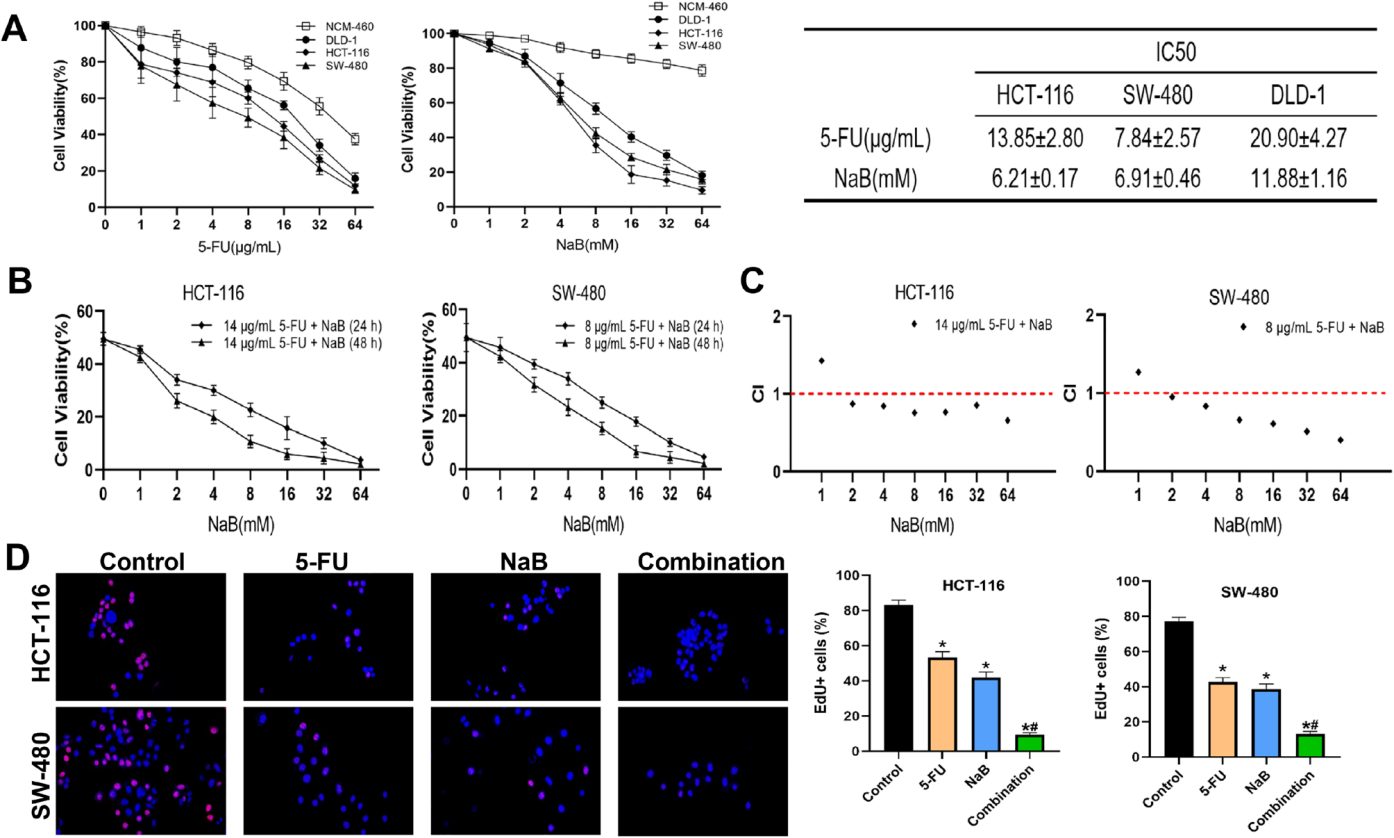
Evaluation of CRC cells’ growth inhibition induced by 5-FU and/or NaB. (**A**) Cells (HCT-116, SW-480, DLD-1, and NCM-460) were treated with 5-FU (0, 1, 2, 4, 8, 16, 32, and 64 µg/mL) or NaB (0, 1, 2, 4, 8, 16, 32, and 64 mM) for 24 h, and cell viability was detected by CCK-8 assay. (**B**) HCT-116 and SW-480 cells were treated with 5-FU and NaB for 24 h or 48 h. (**C**) CompuSyn software was used to define the type of drug-combination effect. (**D**) The proliferation ability of HCT-116 and SW-480 cells was detected by EdU assay; HCT-116 cells were treated with control, 14 µg/mL 5-FU, 6 mM NaB, or 14 µg/mL 5-FU + 6 mM NaB; SW-480 cells were treated with control, 8 µg/mL 5-FU, 7 mM NaB, or 8 µg/mL 5-FU + 7 mM NaB; quantitative analysis of EdU-positive cells (original magnification: ×200). *p < 0.05 vs the control. ^#^*p* < 0.05 vs the 5-FU. All data are shown as the mean ± SD from three independent experiments.

The effects of NaB and 5-FU on cell proliferation were detected by CCK8 and EdU assays. Different concentrations of NaB or 5-FU were administered to CRC and NCM-460 cells for 24 h. All 4 cell lines were differentially sensitive to 5-FU and NaB, with increasing concentrations significantly inhibiting CRC cell viability (Fig. 1A). NCM-460 did not exhibit significant cytotoxicity when the concentration of NaB was below 32 mM. HCT-116 and SW-480 cells were selected for the subsequent experiments because the 2 cell lines are more sensitive to NaB. Subsequently, HCT-116 and SW-480 cells were subjected to combinations of 5-FU and different concentrations of NaB for 24 or 48 h. Figure 1B illustrates that the combination of NaB and 5-FU significantly impeded the proliferation of CRC cells to a greater degree compared to the sole application of 5-FU, demonstrating a time- and dose-dependent effect. CI values demonstrated the synergistic effects of the application of 5-FU and NaB in suppressing the viability of CRC cells (Fig. 1C). For subsequent experiments, HCT-116 and SW-480 cells were treated with 5-FU and NaB at concentrations close to the IC50 value. Consistently, the EdU results indicated that the cells in the NaB plus 5-FU group had poorer proliferation compared to the control and 5-FU groups (Fig. 1D). Taken together, NaB, as one of HDACis, has a strong synergistic effect with 5-FU in colorectal cancer.

### NaB combined with 5-FU induced ROS-mediated apoptosis in CRC cells

**Figure 2:**
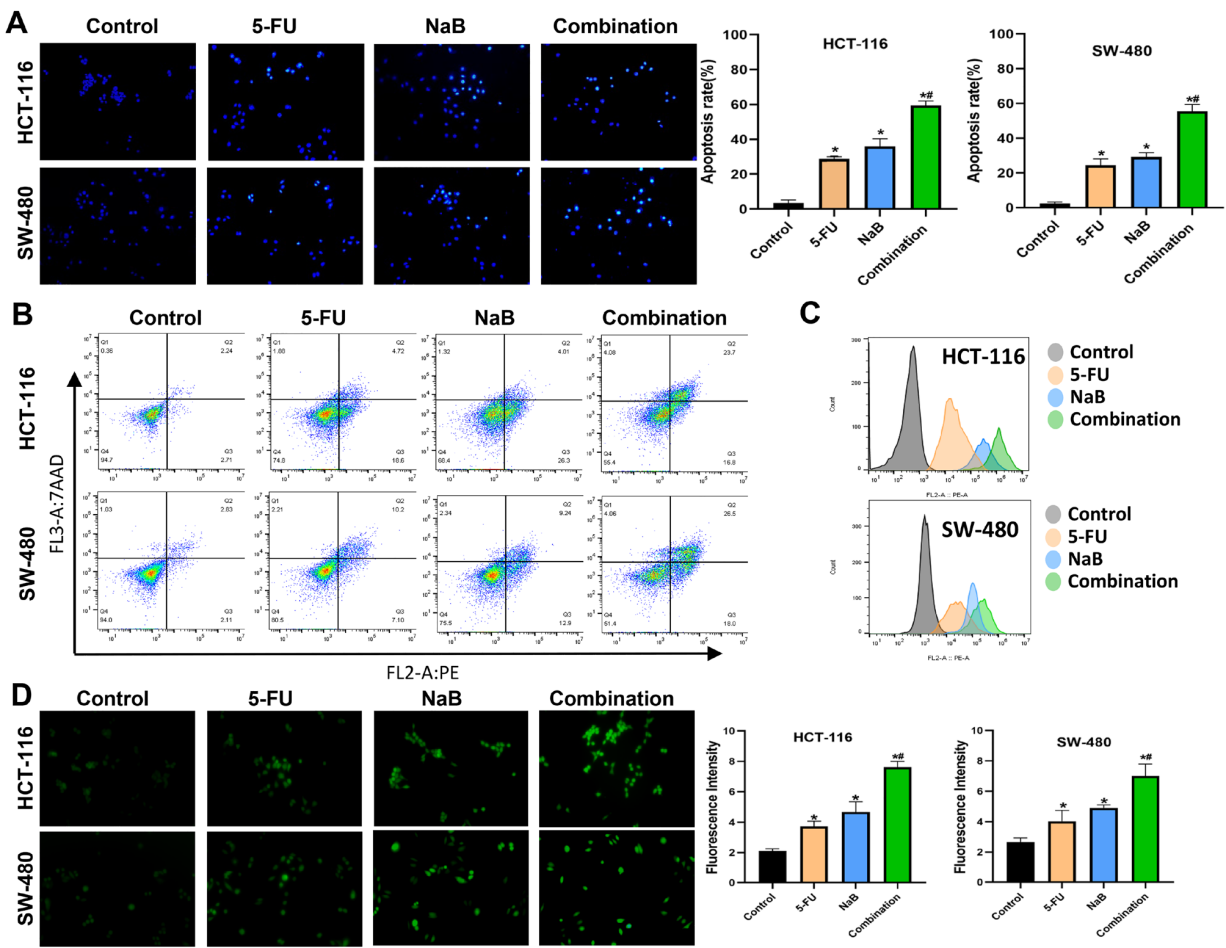
NaB combined with 5-FU promoted ROS-mediated apoptosis in CRC cells. (**A**) The characteristics of apoptotic nuclei in cells treated with specific concentrations (HCT-116 cells were treated with control, 14 µg/mL 5-FU, 6 mM NaB, or 14 µg/mL 5-FU + 6 mM NaB; SW-480 cells were treated with control, 8 µg/mL 5-FU, 7 mM NaB, or 8 µg/mL 5-FU + 7 mM NaB) were observed by Hoechst 33258 staining (original magnification: ×200); quantitative analysis of the apoptosis rate in each group. (**B**) Quantitative flow cytometry measurements of apoptosis in CRC cells. (**C**) Quantitative flow cytometry measurements of ROS in CRC cells. (**D**) ROS was observed via DCFH-DA staining(original magnification: ×200); quantitative analysis of the average fluorescence intensity of ROS in each group. *p < 0.05 vs the control. ^#^*p* < 0.05 vs the 5-FU. All the above data are the mean ± SD from an average of three experiments.

Hoechst 33258 staining coupled with flow cytometry was employed to assess the nuclei of CRC cells following exposure to NaB and/or 5-FU. The results revealed that the combination of NaB and 5-FU induced apoptosis to a larger extent compared to the control and 5-FU groups in CRC cells (Fig. 2A). Annexin V-PE/7AAD staining further corroborated that the combination of NaB and 5-FU induced apoptosis to a larger extent compared to the other treatment modalities (Fig. 2B). Furthermore, Fig. 2C, D illustrate that the NaB combined with 5-FU group of CRC cells exhibited a higher accumulation of ROS (green fluorescence) compared to the control and 5-FU groups. Collectively, NaB combined with 5-FU aggravates the ROS accumulation and apoptosis in CRC cells.

### NaB combined with 5-FU induces mitophagy via the PINK1/Parkin pathway

**Figure 3:**
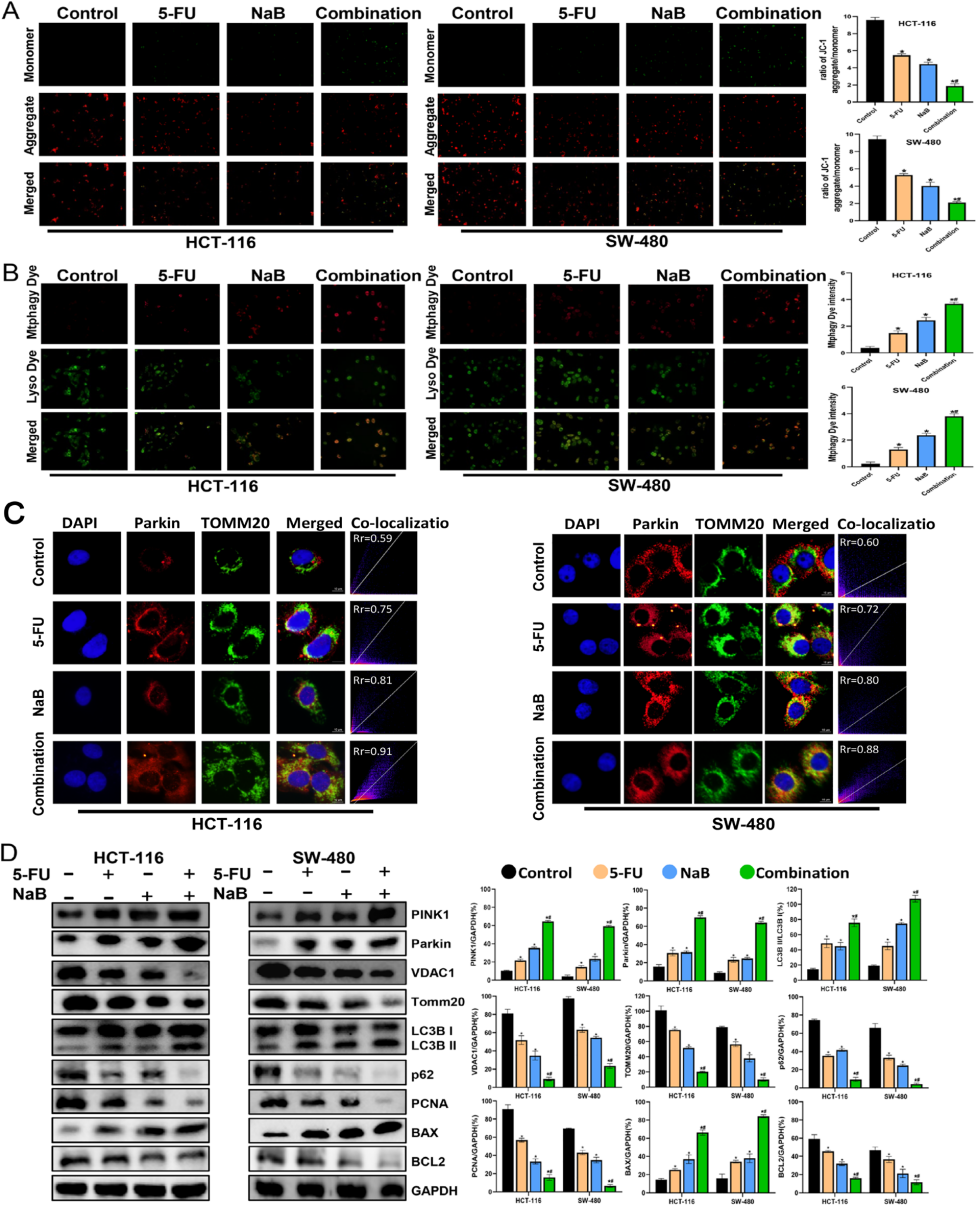
NaB combined with 5-FU promoted mitophagy in CRC Cells. (**A**) MMP was observed via JC-1 staining(original magnification: ×200); quantitative analysis of the MMP in each group. (**B**) Fluorescence intensity of mitochondrial Mtphagy dye and Lyso dye in CRC cells (original magnification: ×400); quantitative analysis of the average fluorescence intensity of mitochondrial Mtphagy dye in each group. (**C**) Co-localization of Parkin with TOMM20 assessed via fluorescence microscopy (original magnification: ×400); co-located scatter plots for quantitative analysis. (**D**) Western blot analysis of PINK1, Parkin, VDAC1, TOMM20, LC3B I, LC3B II, p62, PCNA, BCL2, and BAX was performed in cells; quantitative analysis of the proteins is shown. *p < 0.05 vs the control. ^#^*p* < 0.05 vs the 5-FU. All data are shown as the mean ± SD from three independent experiments.

To explore whether the synergistic action of NaB and 5-FU induces apoptosis in CRC cells through mitochondrial-related signaling, we assessed alterations in MMP levels. The ratio of red-to-green fluorescent intensity in JC-1 staining was utilized to signify alterations in MMP in CRC cells. Elevated red-to-green ratios denote normal membrane potentials. The ratio was lower in the combination group than in the control and 5-FU groups, showing decreased MMP levels(Fig. 3A). The data presented in Fig. 3B indicate that upon treatment with NaB and/or 5-FU, there was partial colocalization of mitophagic vacuoles (stained with Mitophagy Dye) and lysosomes (stained with Lyso Dye) in CRC cells. The intracellular fluorescence intensity of Mitophagy Dye in the combination group was higher than the control and 5-FU groups (Fig. 3B). In addition, immunofluorescence microscopy of drug-treated CRC cells revealed Parkin and TOMM20 co-localization (Fig. 3C).

Western blotting was employed to assess the expression levels of mitophagy pathway-related proteins, providing further validation for the association between the combined treatment and enhanced apoptosis through the PINK1/Parkin pathway. The results revealed increased levels of PINK1, Parkin, and the ratio of LC3B II/LC3B I in the combination group, accompanied by decreased levels of VDAC1, TOMM20, and p62. The magnitude of these changes was more pronounced than that observed with 5-FU alone, as well as compared to the control group (Fig. 3D). Furthermore, the combination group remarkably increased the level of the pro-apoptotic protein BAX and remarkably decreased the levels of anti-apoptotic proteins BCL2 and PCNA (Fig. 3D).

**Figure 4:**
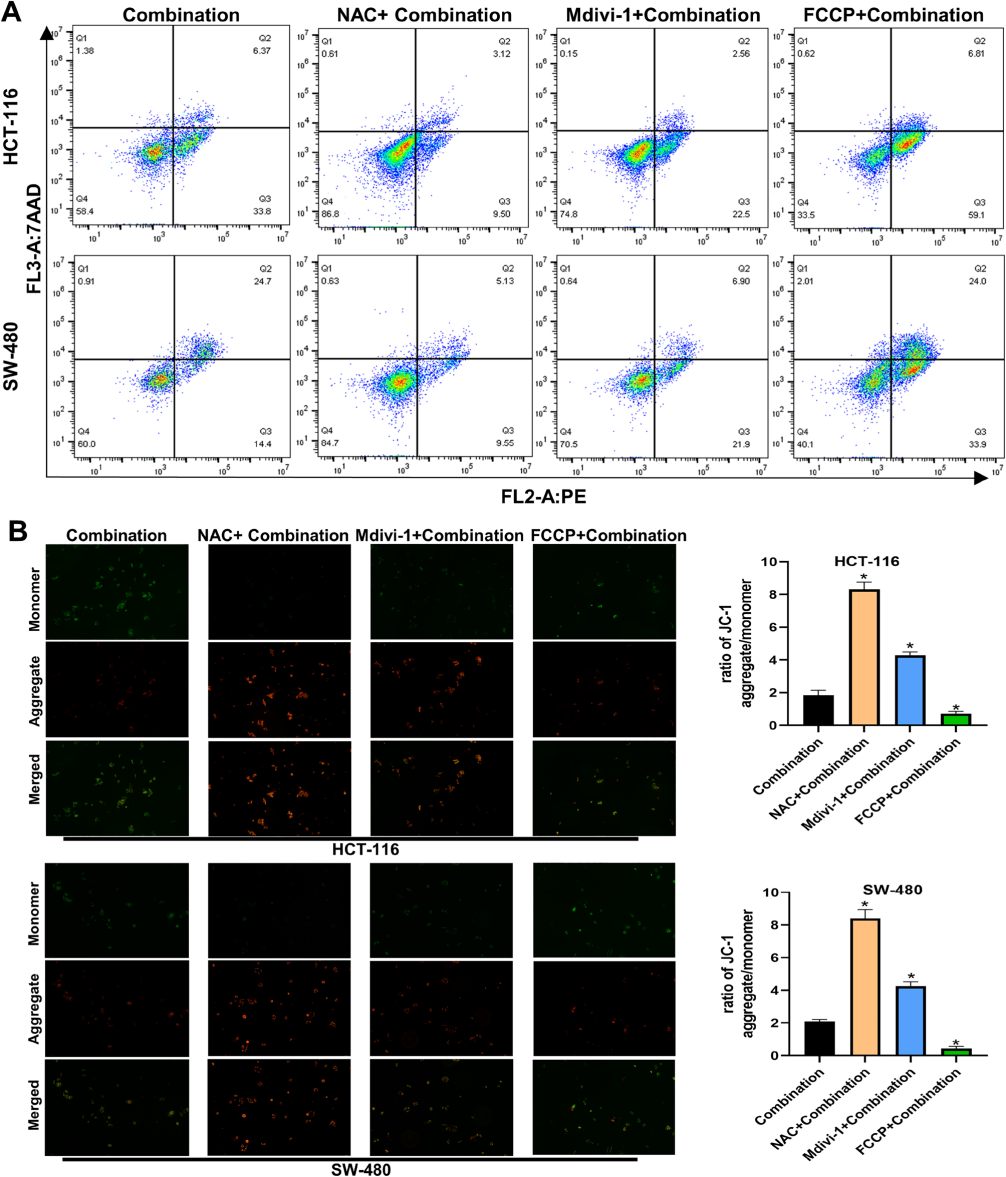
Pretreatment with NAC (10 µm), Mdivi-1 (10 µm), or FCCP (2 µm) respectively influences the apoptosis and MMP of CRC cells induced by NaB combined with 5-FU (**A**) Quantitative flow cytometry measurements of apoptosis in each group(original magnification: ×200). (**B**) MMP was observed via JC-1 staining(original magnification: ×200); quantitative analysis of the MMP in each group. *p < 0.05 vs the Combination. All data are shown as the mean ± SD from three independent experiments.

**Figure 5:**
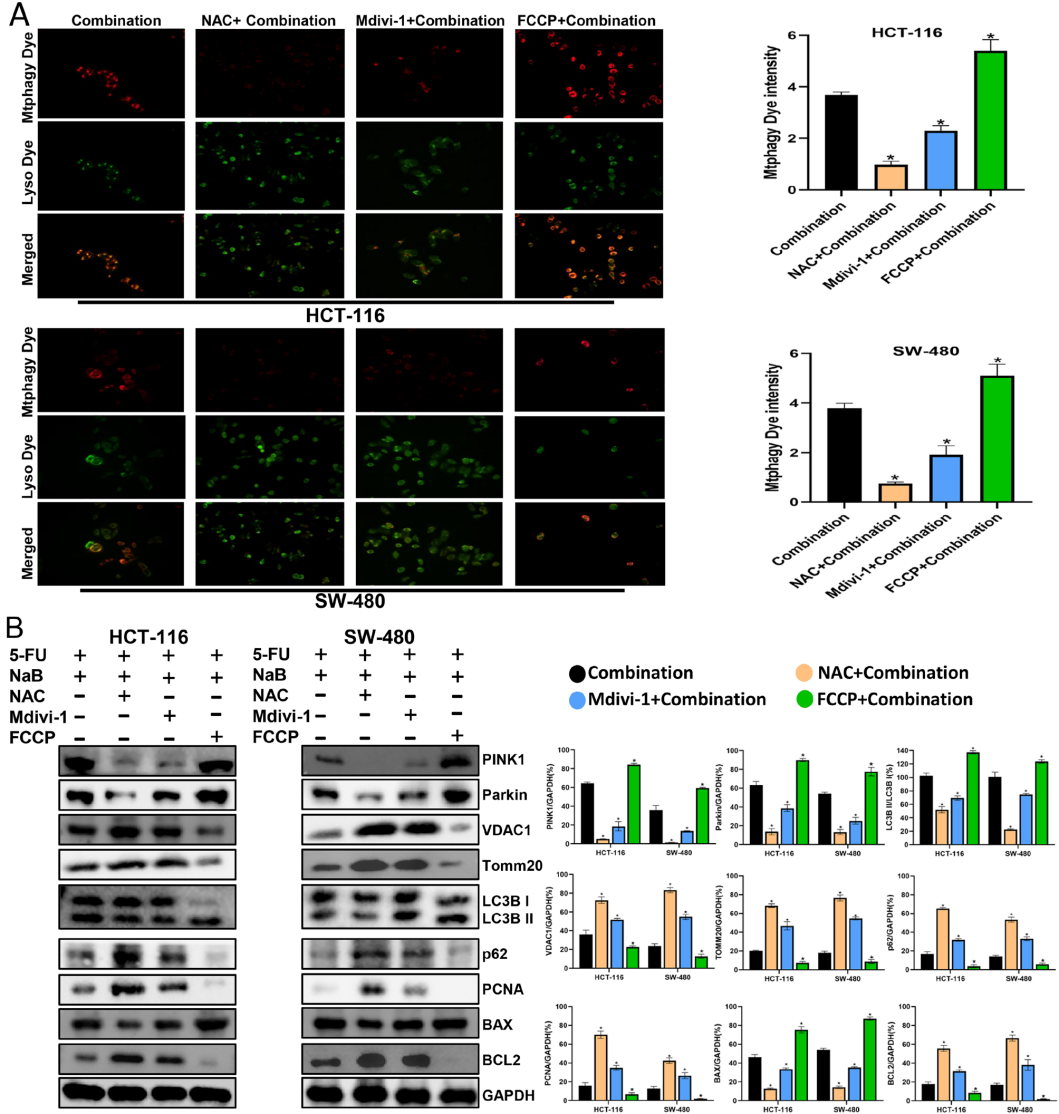
Pretreatment with NAC (10 µm), Mdivi-1 (10 µm), or FCCP (2 µm) respectively influences the mitophagy of CRC cells induced by NaB combined with 5-FU (**A**) Fluorescence intensity of mitochondrial Mtphagy dye and Lyso dye in CRC cells (original magnification: ×400); quantitative analysis of the average fluorescence intensity of mitochondrial Mtphagy Dye in each group. (**B**) Western blot analysis of PINK1, Parkin, VDAC1, TOMM20, LC3B I, LC3B II, p62, PCNA, BCL2, and BAX was performed in cells; quantitative analysis of the proteins is shown. *p < 0.05 vs the combination. All data are shown as the mean ± SD from three independent experiments.

To articulate the molecular mechanisms underlying the heightened apoptosis and mitophagy through the PINK1/Parkin pathway, a ROS inhibitor (NAC), mitophagy inhibitor (Mdivi-1), and mitophagy promoter (FCCP) were employed for a 2-h pretreatment of CRC cells before the administration of the combined drugs. As shown in Fig. 4A, NAC significantly downregulated the level of apoptosis, Mdivi-1 partially downregulated the level of apoptosis and FCCP upregulated the level of apoptosis in CRC cells. Meanwhile, NAC significantly reversed the decrease in MMP, Mdivi-1 partially reversed the decrease in MMP and FCCP increased the loss of MMP induced by the combination (Fig. 4B). In addition, Fig. 5A shows that the level of mitophagy was significantly decreased (NAC group), partially decreased (Mdivi-1 group) or increased (FCCP group). After pretreatment of CRC cells with NAC or Mdivi-1, the levels of PINK1, Parkin, BAX and the ratio of LC3B II/LC3B I were markedly downregulated, while the levels of VDAC1, TOMM20, p62, PCNA and BCL2 were markedly decreased (Fig. 5B). Instead, FCCP further expanded the alterations in the aforementioned protein levels caused by the combined drugs. All these findings suggest that NaB modulates ROS-mediated mitophagy and cytotoxicity through the PINK1/Parkin pathway.

### Anti-tumor effects of NaB and 5-FU on CRC in vivo

**Figure 6:**
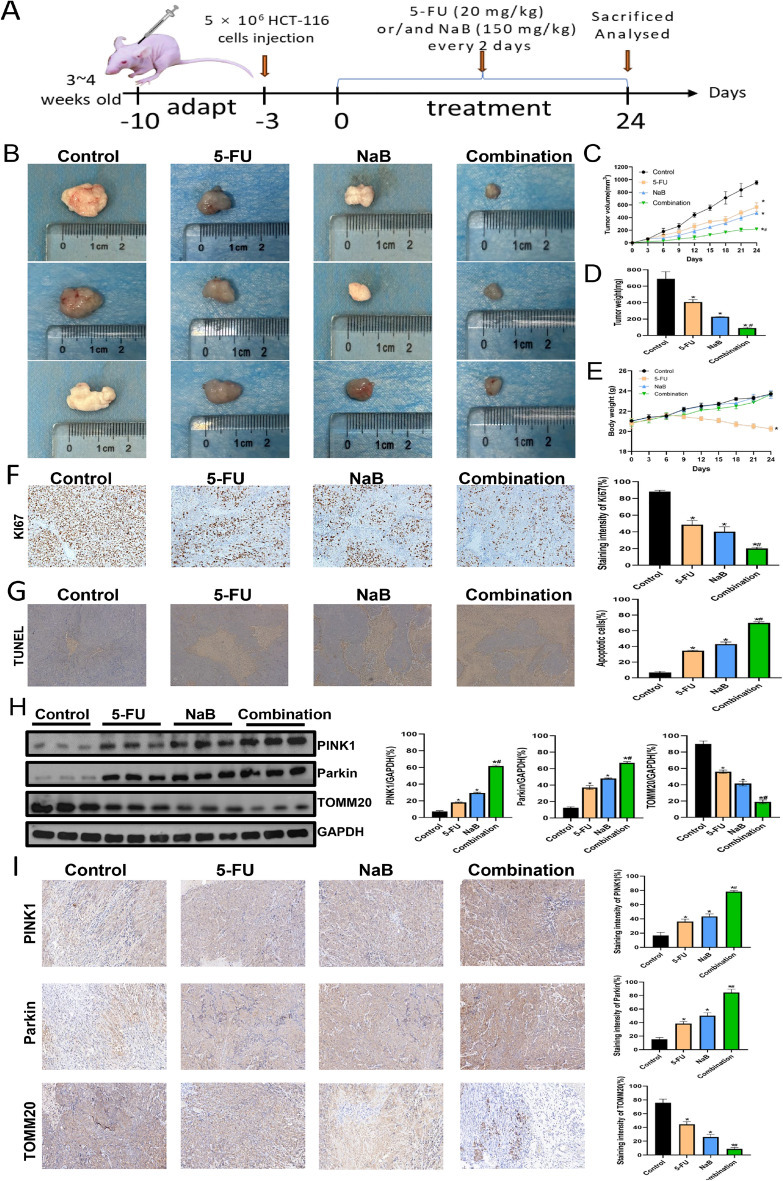
Anti-tumor effects of NaB (150 mg/kg) and 5-FU (20 mg/kg) on HCT-116 cells in vivo. (**A**) Schematic view of the experimental procedures of the xenograft tumor model in nude mice. (**B**) Morphology of the subcutaneous implanted tumor. (**C**) Mean tumor volume at each time point. (**D**) The tumor weight was obtained at the end of the experiment. (**E**) Mean body weight at each time point. (**F**) Immunohistochemical detection of Ki67 expression (original magnification: ×200) and quantitative analysis of Ki67 staining intensity. (**G**) Apoptotic cells were detected in tumor tissue by TUNEL assay (original magnification: ×100); quantitative analysis of apoptotic cells. (**H**) Western blot analysis of PINK1, Parkin, and TOMM20 was performed; quantitative analysis of the proteins is shown. (**I**) Immunohistochemical detection of PINK1, Parkin, and TOMM20 expression (original magnification: ×200) and quantitative analysis of PINK1, Parkin and TOMM20 staining intensity. *p < 0.05 vs the control. ^#^*p* < 0.05 vs the 5-FU. All data are shown as the mean ± SD from three independent experiments.

We conducted an in vivo investigation to elucidate the impact of NaB and/or 5-FU on tumor growth(Fig. 6A). In comparison to the control group, all treatment cohorts exhibited suppressed tumor growth in vivo, evidenced by significantly reduced tumor volume and weight; the most pronounced effect was observed in the combination group (Fig. 6B–D). However, 5-FU-treated nude mice showed a significant decrease in body weight (Fig. 6E). Additionally, as shown in Table 1, we evaluated the hepatic and renal functions of nude mice after treatment with drugs, and 5-FU-treated nude mice had slightly impaired liver and kidney functions, but no significant differences compared to other groups (*p* > 0.05). The TUNEL assay and IHC staining were conducted on tumor tissues isolated from mice. Ki67 staining intensity was most pronounced in the control group (Fig. 6F). In contrast, the TUNEL assay revealed the highest level of apoptosis observed in the combination group (Fig. 6G). Finally, we detected the levels of PINK1, Parkin, and TOMM20 in tumor tissue. The levels of PINK1 and Parkin were found to be significantly elevated in the combination group, while the levels of TOMM20 were observed to be significantly reduced (Fig. 6H, I). In conclusion, these results provide further confirmation that NaB can enhance the anti-tumor efficacy of 5-FU against CRC cells by activating the PINK1/Parkin signaling pathway.

**Table 1:** Effect of NaB combined with 5-FU or alone on hepatic and renal function.

Group	ALT(U/L)	AST(U/L)	Urea(µmol/L)	Cr(µmol/L)
Control	31.60 ± 1.76	142.00 ± 6.81	7.59 ± 0.63	13.18 ± 1.19
5-FU	33.95 ± 2.65	150.60 ± 7.98	9.83 ± 0.99	20.76 ± 2.58
NaB	32.76 ± 2.20	142.80 ± 8.87	8.09 ± 0.82	15.15 ± 1.29
Combination	33.08 ± 1.85	146.80 ± 6.91	8.51 ± 0.40	15.49 ± 2.00

Data are presented as the mean ± standard deviation, with n = 6 mice/group. There were no differences in the ALT, AST, Urea, and Cr levels among all groups (*p* > 0.05).

### Effects of the combination of NaB and 5-FU on alterations in the intestinal flora

**Figure 7:**
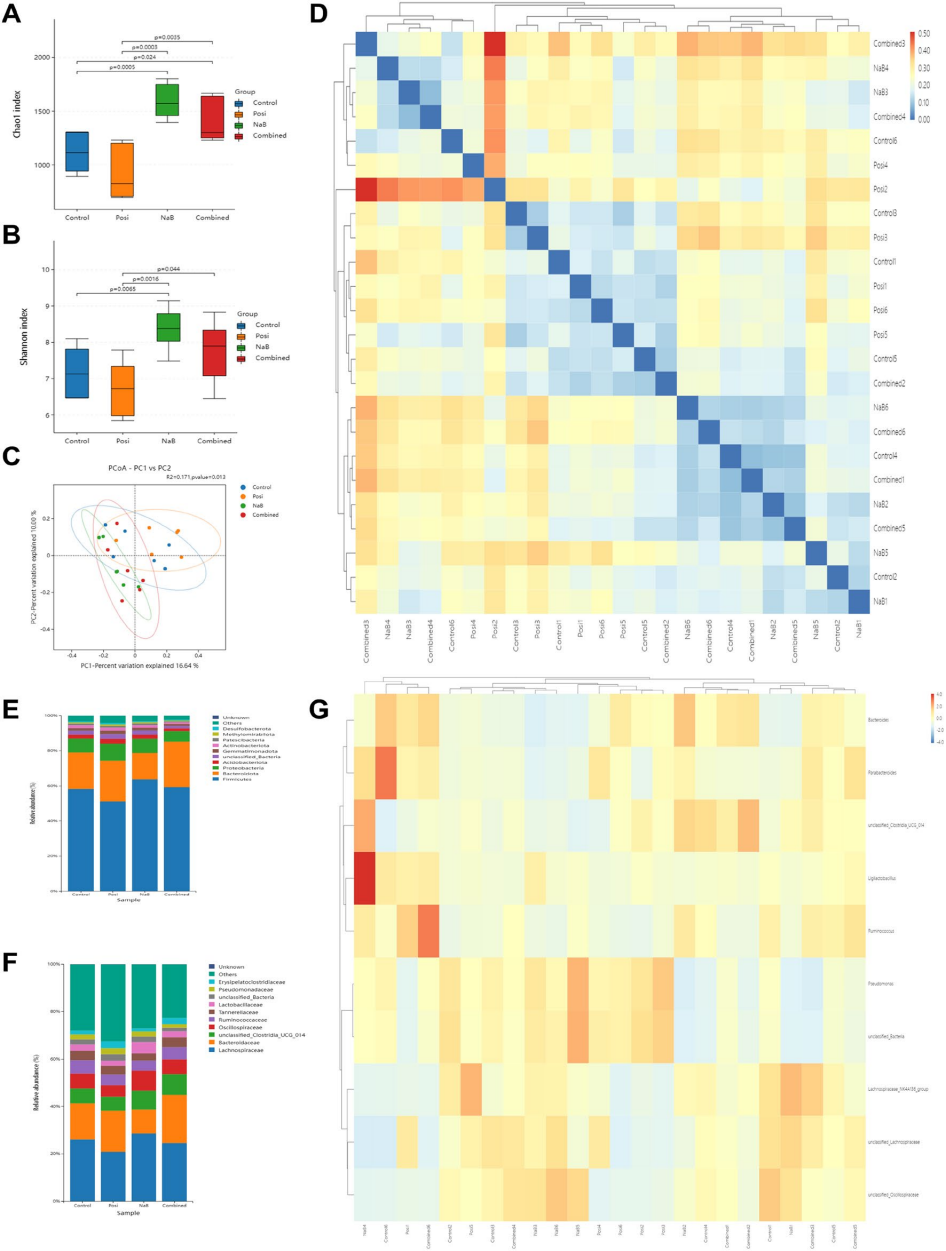
Effects of the combination of NaB (150 mg/kg) and 5-FU (20 mg/kg) on the intestinal flora change in faeces. (**A**) Chao1 index of alpha diversity analysis. (**B**) Shannon index of alpha diversity analysis. (**C**) Principal coordination analysis (PCoA) of beta diversity analysis. (**D**) Sample clustering heat map of beta diversity analysis. (**E,F**) Bar plots of bacterial taxa present in the feces at the phylum and family levels based on relative abundance. (**G**) Heatmap of bacterial taxa present in the feces at the genus level based on relative abundance (n = 6 mice per group). All data are shown as the mean ± SD.

Studies have indicated the involvement of the intestinal flora in CRC. Following treatment, 16S rRNA gene sequencing of fecal flora was conducted to assess species diversity. The combination treatment group exhibited increased alpha diversity compared to the group receiving 5-FU alone [Chao1 index (*p* = 0.0035), Shannon index (*p* = 0.044)] (Fig. 7A, B), indicating that the combination treatment enhanced the richness of the intestinal flora. As shown in Fig. 7C, principal coordinates analysis showed clear differences among the four groups(R^2^ = 0.171, *p* = 0.013) and clustering heat map clearly showed that 5-FU group is significantly different from NaB group and combination group (Fig. 7D). The phylum-level composition of the intestinal flora is showed in Figs. 7E. In contrast to the 5-FU group, the combination group displayed an elevated relative abundance of *Firmicutes*, accompanied by a reduction in the abundance of *Proteobacteria* and *Actinobacteriota*. The relative abundance of bacteria that produce acetic and butyric acid such as *Lachnospiraceae* and *Oscillospiraceae* increased in the combination group compared to 5-FU group. In addition, the relative abundance of beneficial bacteria *Lactobacillaceae* increased while the relative abundance of harmful bacteria *Pseudomonadaceae* and *Erysipelatoclostridiaceae* decreased in the combination group compared to 5-FU group(Fig. 7F). To elucidate the precise alterations in microbial taxa, a detailed analysis of the cluster heat map at the genus level was conducted. In the NaB and combination groups, a noticeable increase in the abundance of beneficial bacteria was observed, including *Bacteroides*, *LigiLactobacillus*, butyric acid-producing bacteria, and acetic acid-producing bacteria (Fig. 7G). These observations imply that NaB treatment has the potential to alleviate the dysbiosis induced by 5-FU. Moreover, the combined treatment demonstrates enhanced therapeutic efficacy by augmenting the levels of beneficial bacteria.

## Discussion

CRC is the most common gastrointestinal malignancy, and the incidence and mortality of CRC continue to increase in China^31^. CRC cells exhibit a capacity to augment resistance to conventional chemotherapy; thus, novel therapeutic approaches are required to enhance the efficacy of 5-FU and surmount mechanisms of resistance^2^. Recently, Grumetti et al. demonstrated that HDACis can enhance the therapeutic efficacy of 5-FU, potentially reducing their toxicity, and ultimately improving the overall survival of cancer patients^32^. Sodium butyrate (NaB), functioning as an HDACi, has undergone evaluation in clinical trials as one of candidate anticancer drugs to treat human malignancies^33,34^. Despite NaB's demonstrated ability to impede proliferation and induce apoptosis, there exists a dearth of comprehensive data elucidating the regulatory mechanisms governing this intricate process. In this study, NaB was highly cytotoxic to colon cancer cells without causing apparent lethality to normal cells in the CCK8 assay. Furthermore, the safety and effectiveness of NaB were corroborated in the nude mice xenograft model, wherein no notable disparities in hepatic and renal function were discerned between the groups treated with NaB and the control group.

Mitochondria occupy a paramount position among cellular organelles, actively engaging in a multifaceted array of cellular processes. These encompass the modulation of apoptosis and the orchestration of autophagy signaling pathways, the generation of ROS, and the synthesis of fatty acids^35^. Notably, dysfunctional mitochondria are the major source of intracellular ROS, leading to mitochondrial oxidative damage. This damage can cause changes in mitochondrial structure and function, including compromised mitochondrial respiratory capacity, impairments in mitochondrial membrane potential (MMP), and an upsurge in mitochondrial-mediated apoptotic processes^36,37^. Anti-cancer drugs mainly work through the mitochondrial pathway, i.e., the endogenous pathway, to promote apoptosis of tumor cells^38,39^. The present study demonstrated, by Hoechst assay and flow cytometry, a significant pro-apoptotic effect of the combination group on CRC cells. In order to elucidate the underlying pathway governing apoptosis, an assessment of ROS levels was conducted, unveiling a significant elevation in ROS levels within the combination group compared to all other groups. Subsequently, an examination of the JC-1 dye in CRC cells was performed to illustrate the MMP level across each group. It was observed that the accrual of ROS precipitated alterations in the MMP of CRC cells, thereby instigating apoptosis.

Studies have highlighted that the interaction of mitochondrial autophagy and ROS represents a plausible focal spot for investigating the mechanisms underlying cancer suppression^40,41^. Mitophagy stands as a pivotal mechanism within the framework of mitochondrial quality control, specializing in the identification and tagging of impaired or dysfunctional mitochondria for subsequent degradation through the autophagic process^42^. Typically, heightened mitophagy represents an early adaptive response aimed at bolstering cell survival. However, it is imperative to recognize that when mitochondrial damage reaches a tipping point, either due to its overwhelming magnitude or protracted duration, it can precipitate an excessive and aberrant activation of mitophagy. This pathological upsurge in mitophagy can ultimately culminate in cellular demise and tissue damage^43^. Furthermore, it is noteworthy that mitochondria-targeted drugs have been reported to induce an immoderate activation of mitophagy, thereby instigating the suppression of CRC^44^. Notably, NaB has demonstrated the capacity to induce both mitophagy and apoptosis in intestinal epithelial cells and skeletal muscle cells^45,46^. In order to ascertain whether NaB elicits mitophagy in CRC cells, the fluorescence intensity of Mitophagy Dye and Lyso Dye was assessed in CRC cells. Significantly, the level of mitophagy in the combination group surpassed that of the other three groups. Subsequently, the combination group was pretreated with Mdivi-1, FCCP, and NAC, revealing substantial variations in the rate of apoptosis, the levels of ROS, the levels of MMP, and the levels of mitophagy between the four groups. This substantiated our assumption that the combined therapeutic triggers apoptosis in CRC cells through the ROS-Mitophagy pathway.

Ordinarily, PINK1 undergoes a continuous importation process to the inner mitochondrial membrane, facilitated by the translocase of the inner mitochondrial membrane (TIMM)-TOMM complex. Subsequently, PINK1 undergoes cleavage by various proteases, including mitochondrial processing peptidase (MPP), ultimately culminating in its proteasomal degradation^47–49^. However, the reduction of MMP and persistent activation of the mitochondrial permeability transition pore (MPTP) lead to accumulation of PINK1 at the OMM of dysfunctional mitochondria^50^. The accumulation of PINK1 also increased biochemical markers of mitophagy activation such as LC3B-II and Parkin, and reduced TOMM20, VADC1, and p62, indicating enhanced mitophagy^51,52^. The results of immunofluorescence confirmed the co-localization of Parkin and TOMM20 after drug treatment. Subsequently, we assessed the levels of pertinent proteins in the mitophagy pathway with Western blotting to elucidate the involvement of the mitophagy pathway in enhancing apoptosis in CRC cells after combination treatment. The outcomes revealed that NaB plus 5-FU significantly elevated the levels of mitophagy markers and concurrently reduced the levels of proliferation-related proteins (PCNA and BCL2). Moreover, the previously mentioned increases in proteins related to the mitophagy pathway were reversed following pretreatment with NAC and Mdivi-1, or accentuated following pretreatment with FCCP. These data imply that the mitophagic process may, under certain circumstances, elevate ROS levels, thereby prompting the cell to instigate further mitophagy, perpetuating the escalation in ROS levels through a positive feedback loop^53^. Concurrently, the accumulation of ROS amplifies mitochondrial damage, thus serving as one of the factors that initiate the cascade of mitophagy ^22^.

Considering the prebiotic perspective of NaB, we postulated that the combined treatment would be more effective in vivo. The data indeed demonstrated that the combined treatment not only impeded the growth of CRC but also markedly augmented the population of apoptotic cells within tumors. Interestingly, the gut microbiota appears to be involved in various processes in the development of CRC^54^. A healthy gut microbiota in normal mice encompasses a harmonious composition of diverse bacterial classes. However, nude mice with tumors exhibited an aberrant gut microbiota, and chemotherapy conspicuously reduced the species diversity and total bacterial counts in the fecal microbiota^55,56^. In this study, discernible deviations in microbial diversity, relative abundance, and compositional similarity were evident in the 5-FU and NaB-treated group as compared to the control group. 16S rRNA sequencing disclosed that the *Bacteroides*, *LigiLactobacillus*, butyric acid-producing bacteria, and acetic acid-producing bacteria were enriched in fecal from the NaB-treated groups. These bacteria are probiotics and are prospective producers of SCFAs^57^. Consequently, it became evident that treatment with NaB refined the dysbiosis caused by 5-FU and, to a certain extent, enhanced the composition of intestinal flora.

However, this study has certain limitations. We only used small molecule inhibitors and agonists to modulate mitophagy. In the future, we plan to use lentiviral transfection techniques to knock down or overexpress mitophagy-related genes to validate the above results.

## Conclusion

In this study, we explored that NaB combined with 5-FU enhances apoptosis in CRC cells in vitro and in vivo. The specific mechanism might be that NaB enhances ROS-mediated mitophagy to induce apoptosis of CRC cells via PINK1/Parkin pathway. Moreover, the restoration of intestinal flora diversity may also be involved in the process (Supplementary Information).

### Supplementary Information

Supplementary Information.

## Data Availability

The data that support the findings of this study are available from the corresponding author, W.D., upon reasonable request.

## References

[1] Sung H, Ferlay J, Siegel RL, Laversanne M, Soerjomataram I, Jemal A, Bray F (2021). Global cancer statistics 2020: GLOBOCAN estimates of incidence and mortality worldwide for 36 cancers in 185 countries. CA Cancer J. Clin..

[2] Vodenkova S, Buchler T, Cervena K, Veskrnova V, Vodicka P, Vymetalkova V (2020). 5-fluorouracil and other fluoropyrimidines in colorectal cancer: Past, present and future. Pharmacol. Ther..

[3] Cai B, Pan J, Chen H, Chen X, Ye Z, Yuan H, Sun H, Wan P (2021). Oyster polysaccharides ameliorate intestinal mucositis and improve metabolism in 5-fluorouracil-treated S180 tumour-bearing mice. Carbohydr. Polym..

[4] Hamouda N, Sano T, Oikawa Y, Ozaki T, Shimakawa M, Matsumoto K, Amagase K, Higuchi K, Kato S (2017). Apoptosis, dysbiosis and expression of inflammatory cytokines are sequential events in the development of 5-fluorouracil-induced intestinal mucositis in mice. Basic Clin. Pharmacol. Toxicol..

[5] Wardill HR, Bowen JM, Gibson RJ (2014). New pharmacotherapy options for chemotherapy-induced alimentary mucositis. Expert Opin. Biol. Ther..

[6] Liu L, Liu Z, Li H, Cao Z, Li W, Song Z, Li X, Lu A, Lu C, Liu Y (2020). Naturally occurring TPE-CA maintains gut microbiota and bile acids homeostasis via FXR signaling modulation of the liver–gut axis. Front. Pharmacol..

[7] Sanna S, van Zuydam NR, Mahajan A, Kurilshikov A, Vich Vila A, Vosa U, Mujagic Z, Masclee AAM, Jonkers D, Oosting M, Joosten LAB, Netea MG, Franke L, Zhernakova A, Fu J, Wijmenga C, McCarthy MI (2019). Causal relationships among the gut microbiome, short-chain fatty acids and metabolic diseases. Nat. Genet..

[8] Wang RX, Lee JS, Campbell EL, Colgan SP (2020). Microbiota-derived butyrate dynamically regulates intestinal homeostasis through regulation of actin-associated protein synaptopodin. Proc. Natl. Acad. Sci. USA.

[9] Wang S, Deng W, Li F, Xiang L, Lv P, Chen Y (2023). Treatment with butyrate alleviates dextran sulfate sodium and *Clostridium difficile*-induced colitis by preventing activity of Th17 cells via regulation of SIRT1/mTOR in mice. J. Nutr. Biochem..

[10] Xue D, Cheng Y, Pang T, Kuai Y, An Y, Wu K, Li Y, Lai M, Wang B, Wang S (2023). Sodium butyrate alleviates deoxynivalenol-induced porcine intestinal barrier disruption by promoting mitochondrial homeostasis via PCK2 signaling. J. Hazard Mater..

[11] Guerriero JL, Sotayo A, Ponichtera HE, Castrillon JA, Pourzia AL, Schad S, Johnson SF, Carrasco RD, Lazo S, Bronson RT, Davis SP, Lobera M, Nolan MA, Letai A (2017). Class IIa HDAC inhibition reduces breast tumours and metastases through anti-tumour macrophages. Nature.

[12] Iannelli F, Roca MS, Lombardi R, Ciardiello C, Grumetti L, De Rienzo S, Moccia T, Vitagliano C, Sorice A, Costantini S, Milone MR, Pucci B, Leone A, Di Gennaro E, Mancini R, Ciliberto G, Bruzzese F, Budillon A (2020). Synergistic antitumor interaction of valproic acid and simvastatin sensitizes prostate cancer to docetaxel by targeting CSCs compartment via YAP inhibition. J. Exp. Clin. Cancer Res..

[13] Wang Z, Shu W, Zhao R, Liu Y, Wang H (2023). Sodium butyrate induces ferroptosis in endometrial cancer cells via the RBM3/SLC7A11 axis. Apoptosis.

[14] Xiao X, Xu Y, Chen H (2020). Sodium butyrate-activated TRAF6-TXNIP pathway affects A549 cells proliferation and migration. Cancer Med..

[15] Wang F, Wu H, Fan M, Yu R, Zhang Y, Liu J, Zhou X, Cai Y, Huang S, Hu Z, Jin X (2020). Sodium butyrate inhibits migration and induces AMPK-mTOR pathway-dependent autophagy and ROS-mediated apoptosis via the miR-139-5p/Bmi-1 axis in human bladder cancer cells. FASEB J..

[16] Chen M, Jiang W, Xiao C, Yang W, Qin Q, Mao A, Tan Q, Lian B, Wei C (2020). Sodium butyrate combined with docetaxel for the treatment of lung adenocarcinoma A549 cells by targeting Gli1. Onco Targets Ther..

[17] Qin X, Xu Y, Peng S, Qian S, Zhang X, Shen S, Yang J, Ye J (2020). Sodium butyrate opens mitochondrial permeability transition pore (MPTP) to induce a proton leak in induction of cell apoptosis. Biochem. Biophys. Res. Commun..

[18] Salimi V, Shahsavari Z, Safizadeh B, Hosseini A, Khademian N, Tavakoli-Yaraki M (2017). Sodium butyrate promotes apoptosis in breast cancer cells through reactive oxygen species (ROS) formation and mitochondrial impairment. Lipids Health Dis..

[19] Bock FJ, Tait SWG (2020). Mitochondria as multifaceted regulators of cell death. Nat. Rev. Mol. Cell Biol..

[20] Litvak DA, Hwang KO, Evers BM, Townsend CM (2000). Induction of apoptosis in human gastric cancer by sodium butyrate. Anticancer Res..

[21] Katayama H, Hama H, Nagasawa K, Kurokawa H, Sugiyama M, Ando R, Funata M, Yoshida N, Homma M, Nishimura T, Takahashi M, Ishida Y, Hioki H, Tsujihata Y, Miyawaki A (2020). Visualizing and modulating mitophagy for therapeutic studies of neurodegeneration. Cell.

[22] Zhao M, Wang Y, Li L, Liu S, Wang C, Yuan Y, Yang G, Chen Y, Cheng J, Lu Y, Liu J (2021). Mitochondrial ROS promote mitochondrial dysfunction and inflammation in ischemic acute kidney injury by disrupting TFAM-mediated mtDNA maintenance. Theranostics.

[23] Wang Y, Tang C, Cai J, Chen G, Zhang D, Zhang Z, Dong Z (2018). PINK1/Parkin-mediated mitophagy is activated in cisplatin nephrotoxicity to protect against kidney injury. Cell Death Dis..

[24] Lazarou M, Sliter DA, Kane LA, Sarraf SA, Wang C, Burman JL, Sideris DP, Fogel AI, Youle RJ (2015). The ubiquitin kinase PINK1 recruits autophagy receptors to induce mitophagy. Nature.

[25] Satoh K, Yachida S, Sugimoto M, Oshima M, Nakagawa T, Akamoto S, Tabata S, Saitoh K, Kato K, Sato S, Igarashi K, Aizawa Y, Kajino-Sakamoto R, Kojima Y, Fujishita T, Enomoto A, Hirayama A, Ishikawa T, Taketo MM, Kushida Y, Haba R, Okano K, Tomita M, Suzuki Y, Fukuda S, Aoki M, Soga T (2017). Global metabolic reprogramming of colorectal cancer occurs at adenoma stage and is induced by MYC. Proc. Natl. Acad. Sci. USA.

[26] Sun X, Shu Y, Ye G, Wu C, Xu M, Gao R, Huang D, Zhang J (2022). Histone deacetylase inhibitors inhibit cervical cancer growth through Parkin acetylation-mediated mitophagy. Acta Pharm. Sin. B.

[27] Wang L, Qi H, Tang Y, Shen HM (2020). Post-translational modifications of key machinery in the control of mitophagy. Trends Biochem. Sci..

[28] Di Sante G, Pestell TG, Casimiro MC, Bisetto S, Powell MJ, Lisanti MP, Cordon-Cardo C, Castillo-Martin M, Bonal DM, Debattisti V, Chen K, Wang L, He X, McBurney MW, Pestell RG (2015). Loss of Sirt1 promotes prostatic intraepithelial neoplasia, reduces mitophagy, and delays PARK2 translocation to mitochondria. Am. J. Pathol..

[29] Song SB, Jang SY, Kang HT, Wei B, Jeoun UW, Yoon GS, Hwang ES (2017). Modulation of mitochondrial membrane potential and ROS generation by nicotinamide in a manner independent of SIRT1 and mitophagy. Mol. Cells.

[30] Sun F, Jiang X, Wang X, Bao Y, Feng G, Liu H, Kou X, Zhu Q, Jiang L, Yang Y (2019). Vincristine ablation of Sirt2 induces cell apoptosis and mitophagy via Hsp70 acetylation in MDA-MB-231 cells. Biochem. Pharmacol..

[31] Li N, Lu B, Luo C, Cai J, Lu M, Zhang Y, Chen H, Dai M (2021). Incidence, mortality, survival, risk factor and screening of colorectal cancer: A comparison among China, Europe, and northern America. Cancer Lett..

[32] Grumetti L, Lombardi R, Iannelli F, Pucci B, Avallone A, Di Gennaro E, Budillon A (2022). Epigenetic approaches to overcome fluoropyrimidines resistance in solid tumors. Cancers (Basel).

[33] Lane AA, Chabner BA (2009). Histone deacetylase inhibitors in cancer therapy. J. Clin. Oncol..

[34] Ma X, Ezzeldin HH, Diasio RB (2009). Histone deacetylase inhibitors: Current status and overview of recent clinical trials. Drugs.

[35] Pfanner N, Warscheid B, Wiedemann N (2019). Mitochondrial proteins: From biogenesis to functional networks. Nat. Rev. Mol. Cell Biol..

[36] Friedman JR, Nunnari J (2014). Mitochondrial form and function. Nature.

[37] Yang YY, Gong DJ, Zhang JJ, Liu XH, Wang L (2019). Diabetes aggravates renal ischemia-reperfusion injury by repressing mitochondrial function and PINK1/Parkin-mediated mitophagy. Am. J. Physiol. Renal Physiol..

[38] Green DR (2022). The mitochondrial pathway of apoptosis: Part I: MOMP and beyond. Cold Spring Harb. Perspect. Biol..

[39] Green DR (2022). The mitochondrial pathway of apoptosis Part II: The BCL-2 protein family. Cold Spring Harb. Perspect. Biol..

[40] Panigrahi DP, Praharaj PP, Bhol CS, Mahapatra KK, Patra S, Behera BP, Mishra SR, Bhutia SK (2020). The emerging, multifaceted role of mitophagy in cancer and cancer therapeutics. Semin. Cancer Biol..

[41] Liu M, Fan Y, Li D, Han B, Meng Y, Chen F, Liu T, Song Z, Han Y, Huang L, Chang Y, Cao P, Nakai A, Tan K (2021). Ferroptosis inducer erastin sensitizes NSCLC cells to celastrol through activation of the ROS-mitochondrial fission-mitophagy axis. Mol. Oncol..

[42] Palikaras K, Lionaki E, Tavernarakis N (2018). Mechanisms of mitophagy in cellular homeostasis, physiology and pathology. Nat. Cell Biol..

[43] Aggarwal S, Mannam P, Zhang J (2016). Differential regulation of autophagy and mitophagy in pulmonary diseases. Am. J. Physiol. Lung Cell Mol. Physiol..

[44] Boyle KA, Van Wickle J, Hill RB, Marchese A, Kalyanaraman B, Dwinell MB (2018). Mitochondria-targeted drugs stimulate mitophagy and abrogate colon cancer cell proliferation. J. Biol. Chem..

[45] Li X, Wang C, Zhu J, Lin Q, Yu M, Wen J, Feng J, Hu C (2022). Sodium butyrate ameliorates oxidative stress-induced intestinal epithelium barrier injury and mitochondrial damage through AMPK-mitophagy pathway. Oxid. Med. Cell Longev..

[46] Ding Y, Wang P, Li C, Zhang Y, Yang C, Zhou X, Wang X, Su Z, Ming W, Zeng L, Shi Y, Li CJ, Kang X (2023). Sodium butyrate induces mitophagy and apoptosis of bovine skeletal muscle satellite cells through the mammalian target of rapamycin signaling pathway. Int. J. Mol. Sci..

[47] Jin SM, Lazarou M, Wang C, Kane LA, Narendra DP, Youle RJ (2010). Mitochondrial membrane potential regulates PINK1 import and proteolytic destabilization by PARL. J. Cell Biol..

[48] Deas E, Plun-Favreau H, Gandhi S, Desmond H, Kjaer S, Loh SH, Renton AE, Harvey RJ, Whitworth AJ, Martins LM, Abramov AY, Wood NW (2011). PINK1 cleavage at position A103 by the mitochondrial protease PARL. Hum. Mol. Genet..

[49] Zhang C, Wang R, Liu Z, Bunker E, Lee S, Giuntini M, Chapnick D, Liu X (2019). The plant triterpenoid celastrol blocks PINK1-dependent mitophagy by disrupting PINK1's association with the mitochondrial protein TOM20. J. Biol. Chem..

[50] Lazarou M, Jin SM, Kane LA, Youle RJ (2012). Role of PINK1 binding to the TOM complex and alternate intracellular membranes in recruitment and activation of the E3 ligase Parkin. Dev. Cell.

[51] Vives-Bauza C, Zhou C, Huang Y, Cui M, de Vries RL, Kim J, May J, Tocilescu MA, Liu W, Ko HS, Magrane J, Moore DJ, Dawson VL, Grailhe R, Dawson TM, Li C, Tieu K, Przedborski S (2010). PINK1-dependent recruitment of Parkin to mitochondria in mitophagy. Proc. Natl. Acad. Sci. USA.

[52] Shiba-Fukushima K, Inoshita T, Hattori N, Imai Y (2014). PINK1-mediated phosphorylation of Parkin boosts Parkin activity in Drosophila. PLoS Genet..

[53] Schofield JH, Schafer ZT (2021). Mitochondrial reactive oxygen species and mitophagy: A complex and nuanced relationship. Antioxid. Redox Signal.

[54] Tilg H, Adolph TE, Gerner RR, Moschen AR (2018). The intestinal microbiota in colorectal cancer. Cancer Cell.

[55] Yuan X, Xue J, Tan Y, Yang Q, Qin Z, Bao X, Li S, Pan L, Jiang Z, Wang Y, Lou Y, Jiang L, Du J (2021). Albuca bracteate polysaccharides synergistically enhance the anti-tumor efficacy of 5-fluorouracil against colorectal cancer by modulating beta-catenin signaling and intestinal flora. Front. Pharmacol..

[56] Wang C, Yang S, Gao L, Wang L, Cao L (2018). Carboxymethyl pachyman (CMP) reduces intestinal mucositis and regulates the intestinal microflora in 5-fluorouracil-treated CT26 tumour-bearing mice. Food Funct..

[57] Markowiak-Kopec P, Slizewska K (2020). The effect of probiotics on the production of short-chain fatty acids by human intestinal microbiome. Nutrients.

