# Supplementary material for: Microbial metabolite sodium butyrate enhances the anti-tumor efficacy of 5-fluorouracil against colorectal cancer by modulating PINK1/Parkin signaling and intestinal flora
Source: Sci Rep. 2024 Jun 6;14:13063. doi: 10.1038/s41598-024-63993-x (PMC11156851; doi:10.1038/s41598-024-63993-x)
Supplement: Supplementary file 1 — Supplementary Information. [file 41598_2024_63993_MOESM1_ESM.pdf]

Microbial Metabolite Sodium Butyrate Enhances the Anti-tumor Efficacy of 5-fluorouracil Against Colorectal Cancer by Modulating PINK1/Parkin Signaling and Intestinal Flora

Yangbo Li, [†a,b] Pengzhan He, [†a,b] Ying Chen, [†a,b] Jiaming Hu, [a,b] Beiying Deng, [a,b] Chuan Liu, [a,b] Baoping Yu [*a] and Weiguo Dong, [*a]

[a]Department of Gastroenterology, Renmin Hospital of Wuhan University, Wuhan, China

[b]Central Laboratory, Renmin Hospital of Wuhan University, Wuhan, China

[*]Correspondence

Baoping Yu, Department of Gastroenterology, Renmin Hospital of Wuhan University, No. Zhang Zhi-dong Road, Wuhan 430060, Hubei Province, PR China.Email: yubp62@163.com

Weiguo Dong, Department of Gastroenterology, Renmin Hospital of Wuhan University, No. Zhang Zhi-dong Road, Wuhan 430060, Hubei Province, PR China.Email: dongweiguo@whu.edu.cn

[†]Yangbo Li, Pengzhan He, and Ying Chen contributed equally to this study.

Original images of Representative Western blot

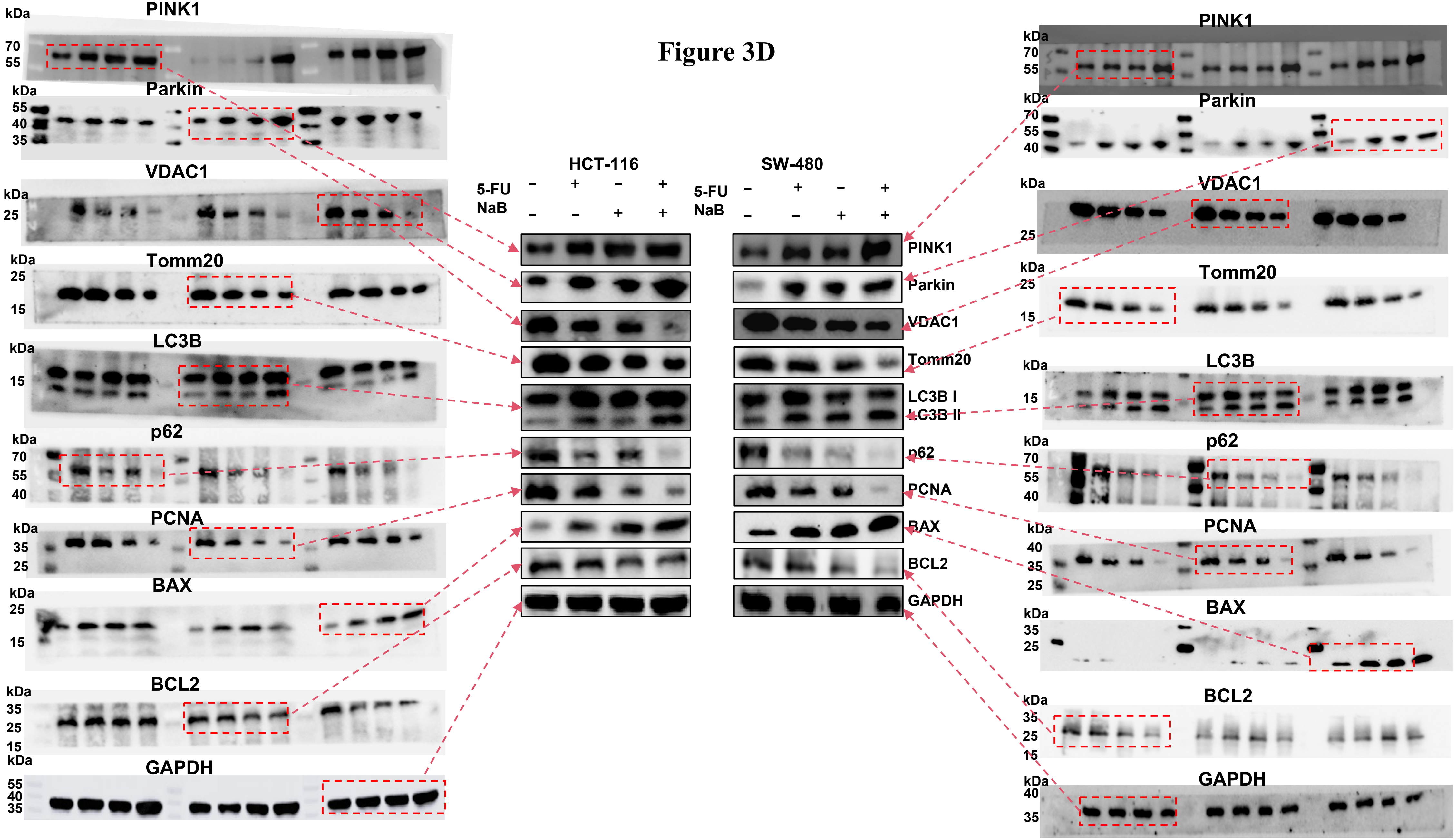

Figure 5B

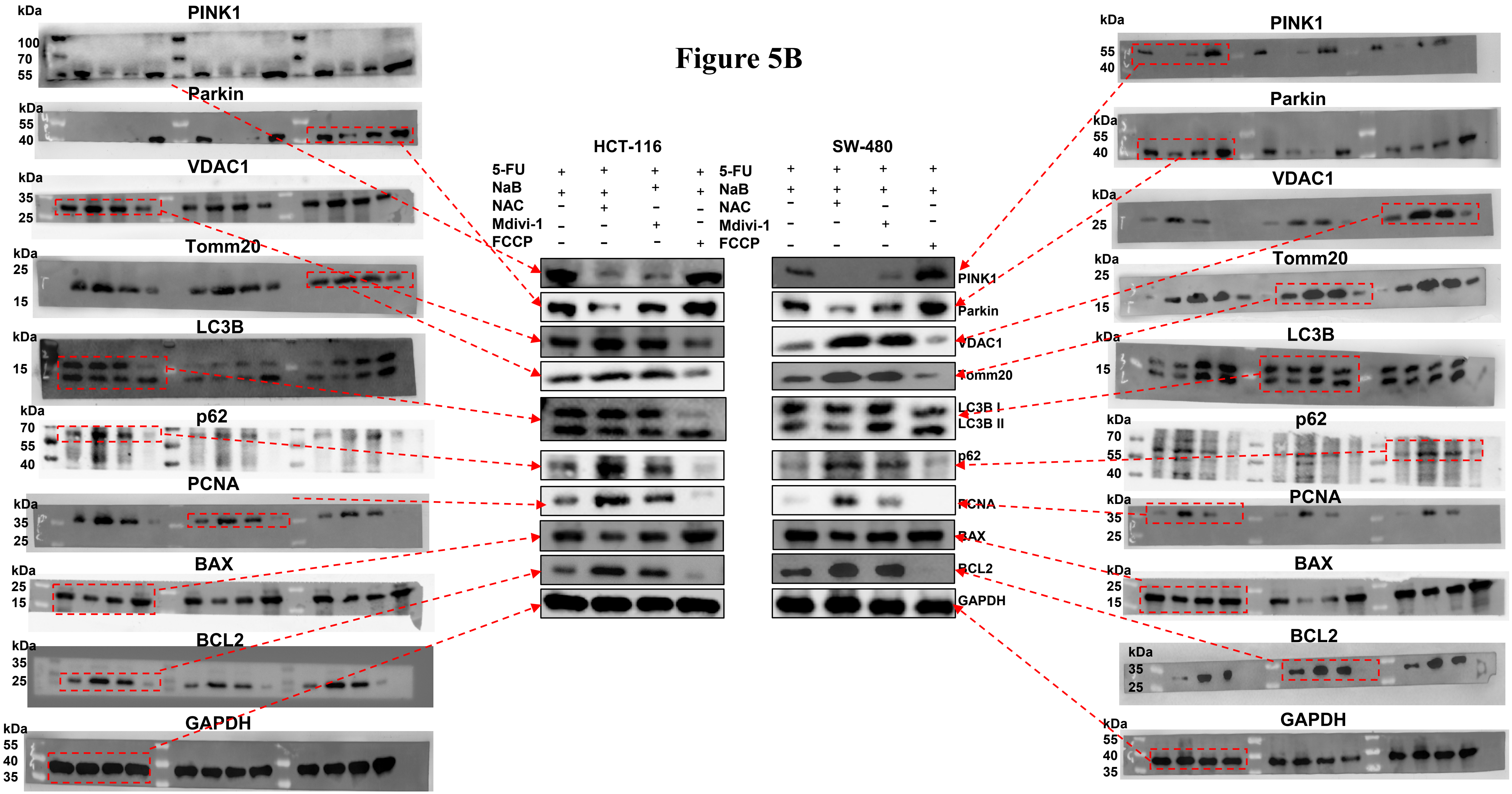
HCT-116
SW-480
5-FU
NaB
NAC
Mdivi-1
FCCP
PINK1
Parkin
VDAC1
Tomm20
LC3B
LC3B I
LC3B II
p62
PCNA
BAX
BCL2
GAPDH
kDa
100
70
55
40
35
25
15

Figure 6H

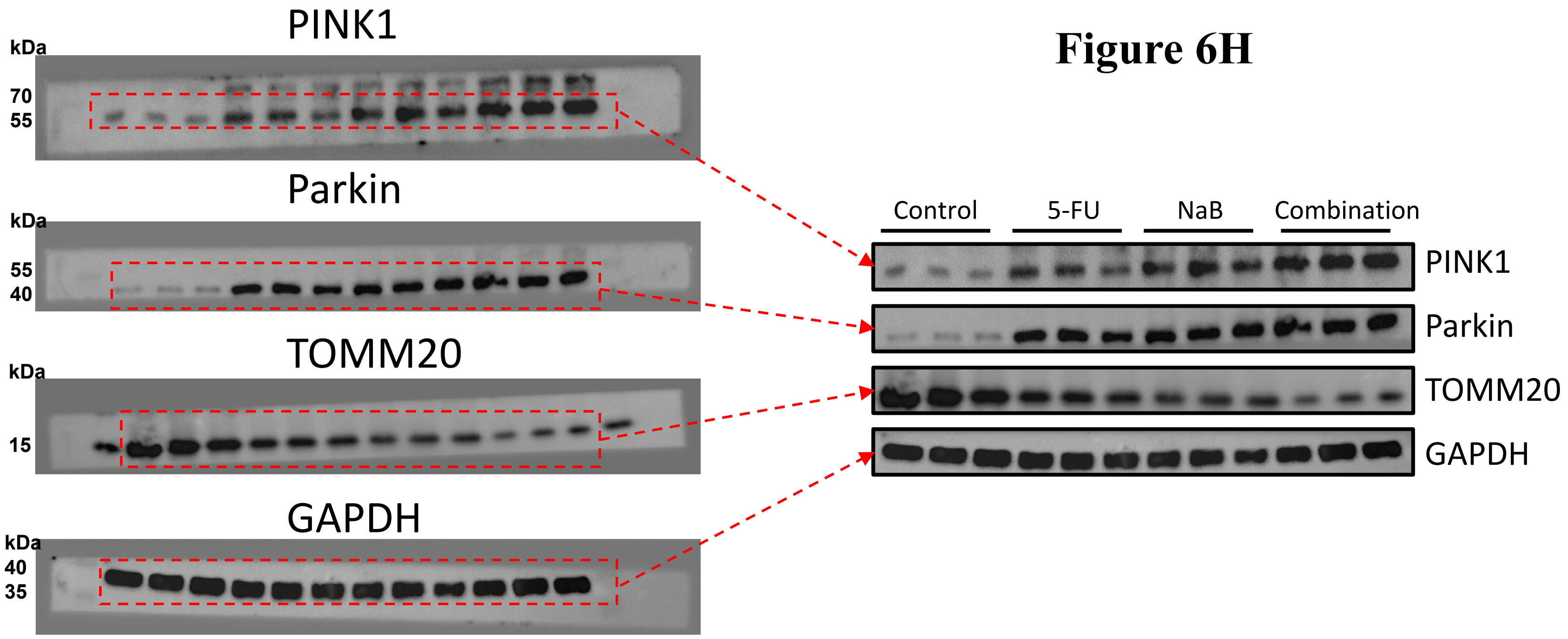
PINK1
kDa
70
55
Parkin
kDa
55
40
TOMM20
kDa
15
GAPDH
kDa
40
35
Control
5-FU
NaB
Combination
PINK1
Parkin
TOMM20
GAPDH
